# Supplementary material for: Listening to Transgender Patients and Their Providers in Non-Metropolitan Spaces: Needs, Gaps, and Patient-Provider Discrepancies
Source: Int J Environ Res Public Health. 2021 Oct 15;18(20):10843. doi: 10.3390/ijerph182010843 (PMC8535616; doi:10.3390/ijerph182010843)
Supplement: Supplementary file 1 [file ijerph-18-10843-s001.zip › ijerph-1365084-supplementary.pdf]

Semi-Structured Interview: Providers

This interview includes questions about your experience at the STITCH conference you attended on April 27th. We want to know more about your experiences at the conference and how to improve the impact of these kinds of events on providers ability to care for trans and non gender binary patients. Before we start the interview I have two brief questions:

- How many "trans health" conferences have you attended including STITCH?
- About how many trans people do you treat as patients? (a few, a good bit, a lot, etc.)

1. Tell me about how you made the decision to attend STITCH? [What influenced you to attend? How did you find out about it? What was *your* goal in attending?] Intent: basic ice breaker to learn more about interviewee
2. What audience(s) do you think STITCH was designed for? Intent: to understand interviewee's perspective on conference and match between conference and interviewee expectations
3. What do you think the goals of STITCH were? Intent: same as #2
4. What sessions/topic discussions do you remember attending at STITCH? [What made these memorable?] Intent: understand effect of conference
5. What did you expect STITCH to be like? [What did you expect to learn?] How was STITCH and/or your learning at STITCH different than your expectations? Intent: Same as #4
6. What are some of the most important things *you* learned at STITCH about – about trans health overall? Trans people? Yourself or your training needs? Intent: Same as #4
7. What do you think motivated *trans people* to attend STITCH? Intent: Same as #4
8. If you were in charge of STITCH which topics would you have included on the agenda? [For trans folks, for providers? What topics should have been covered but were missing from STITCH? What questions remained when you left?] Intent: Identify gaps or problems with conference
9. What are trans folks' top health concerns? Intent: Learn more about provider training needs
10. What are providers' or health care systems' main needs in addressing trans care? What are *your* main needs in addressing trans people's health care? Intent: Same as 9
11. How or how not, are conferences like STITCH, a place for providers to learn about caring for trans people and their health needs? [How or how not, would it help to have something like this as a regular or series event versus a once a year event?] Intent: learn how we can use these events to adequately train providers
12. Is there anything else you would like to tell me about yourself, your training needs or your experience at STITCH? Intent: conclusion

Thank you for taking part in the interview.

Semi-Structured Interview: Community members

This interview includes questions about your experience at the STITCH conference you attended on April 27th. We want to know more about your experiences at the conference and how to improve the impact of these kinds of events on transgender (trans) and gender non binary folks' ability to advocate for themselves in healthcare. Before we start the interview I have two brief questions:

- How many "trans health" conferences have you attended including STITCH?
- Preferred pronoun

13. Tell me about how you made the decision to attend STITCH? [What influenced you to attend? How did you find out about it? What was *your* goal in attending?] Intent: basic ice breaker to learn more about interviewee
14. What audience(s) do you think STITCH was designed for? Intent: to understand interviewee's perspective on conference and match between conference and interviewee expectations
15. What do you think the goals of STITCH were? Intent: same as #2
16. What sessions/topic discussions do you remember attending at STITCH? [What made these memorable?] Intent: understand effect of conference
17. What did you expect STITCH to be like? [What did you expect to learn?] How was STITCH and/or your learning at STITCH different than your expectations? Intent: Same as #4
18. What are some of the most important things *you* learned at STITCH about – trans healthcare overall? Medical providers and practices? Yourself or your needs? Intent: Same as #4
19. What do you think *medical providers* needed to learn at STITCH? Intent: Same as #4
20. If you were in charge of STITCH which topics would you have included on the agenda? [For trans folks, for providers? What topics should have been covered but were missing from STITCH?] Intent: Identify gaps or problems with conference
21. What are trans folks' top health concerns? What are your top health concerns? Intent: Learn more about trans health needs
22. What do trans folks need to be able to advocate for themselves in health care and with medical providers? Intent: Same as 9
23. How or how not, are conferences like STITCH, a place for trans people to learn about their health and how to advocate for their health needs? Intent: learn how we can use these events to empower trans folks
24. Is there anything else you would like to tell me about yourself, your health needs or your experience at STITCH? Intent: conclusion

Thank you for taking part in the interview.
